# Preoperative prognostic assessment using intratumoral and peritumoral adipose tissue radiomics derived from contrast-enhanced CT in cT3–4 gastric cancer

**DOI:** 10.3389/fonc.2025.1631979

**Published:** 2025-10-09

**Authors:** Chao Lu, Donggang Pan, Xiuhong Shan, Rao Dai, Bowen Liu, Zhixuan Wang, Xiaoxiao Wang

**Affiliations:** Department of Medical Imaging, Jiangsu University Affiliated People’s Hospital (Zhenjiang First People’s Hospital), Zhenjiang, Jiangsu, China

**Keywords:** gastric cancer, contrast-enhanced computed tomography, radiomics, prognosis, peritumoral adipose tissue

## Abstract

**Purpose:**

Exploring the value of contrast-enhanced computed tomography (CECT) based radiomics features from intratumoral and peritumoral adipose tissue (PAT) in predicting early recurrence (ER)after gastrectomy in patients with cT3–4 gastric cancer (GC).

**Materials and methods:**

This retrospective study involved patients with cT3–4 GC who underwent preoperative CECT. The radiomics features of tumor and PAT were separately extracted from the CT venous phase images using the Pyradiomics package. The radiomic score (radscore) was computed for each patient by integrating LASSO regression-selected radiomic features, weighted according to their respective coefficients. The GC location, longest diameter, maximum thickness, cT stage and cN stage determined by preoperative CT were also evaluated. Univariate and bivariate analyses using the Cox regression model were performed to evaluate factors affecting ER. The Kaplan-Meier method was used for the analysis of ER-free survival.

**Results:**

A total of 184 consecutive cT3–4 GC patients were enrolled in this study. Bivariate Cox regression analyses demonstrated that radscore and cT stage emerged as independent predictors of ER in all parameters. Radscore-based stratification showed a marked difference in the ER rates between high-risk patients (radscore ≥ -0.66) and low-risk patients (65.9% vs. 3.2%; log-rank p<0.001). Similarly, cT4 stage patients had markedly higher ER rates than cT3 stage patients (53.5% vs. 22.1%; log-rank p<0.001).

**Conclusion:**

The integrated radscore combining intratumoral and PAT features emerged as an independent prognostic predictor for ER in cT3–4 GC, offering quantitative biomarkers to optimize neoadjuvant therapy selection and postoperative surveillance intensity.

## Introduction

Gastric cancer (GC) ranks among the most prevalent malignancies globally, with the 5th and 5rd highest incidence and mortality rates among malignant tumours in the world, respectively (1). The majority (60-70%) of GC-related deaths are due to recurrence, which often occurs within the first 2 years after gastrectomy (2), which is called early recurrence (ER). Early prediction of the prognosis of gastric cancer and the development of individualised treatment plans can improve the prognosis of gastric cancer.

The clinical TNM staging system (cTNM) plays a pivotal role in therapeutic planning and risk stratification for gastric cancer. CT serves as the primary imaging modality for evaluating tumor invasiveness (T-stage) and lymph node metastasis (N-stage). While CT-based assessment of serosal infiltration and nodal involvement demonstrates strong prognostic value (3, 4), its clinical utility is constrained by substantial inter-observer variability, particularly in differentiating T3 (subserosal invasion) from T4 (serosal penetration) lesions. This diagnostic challenge arises because both stages frequently present radiologically as tumors involving the full gastric wall thickness (5).Growing evidence highlights marked prognostic variability among gastric cancer patients with identical cT and cN stages, even in the absence of distant metastases (6). This evidence gap highlights the need to identify additional independent imaging biomarkers to improve risk stratification frameworks to facilitate precision treatment approaches.

Radiomics, which extracts and analyzes high-dimensional quantitative features from medical images, has shown promise in predicting GC prognosis (7). Previous studies have shown that PAT information can accurately assess plasma membrane invasion and reliably predict response to neoadjuvant chemotherapy (8, 9). This is a caution that peritumoural adipose tissue contains important biological information that we have overlooked. Thus, We hypothesised that a combination of tumour and PAT radiomics might demonstrate prognostic significance in GC. This study aims to assess the feasibility of these radiomic features derived from contrast-enhanced CT (CECT) for preoperative ER risk stratification in cT3–4 GC patients.

## Materials and methods

### Patient characteristics

This retrospective study was approved by the Institutional Review Board of our hospital. The inclusion criteria were as follows: (1) a pathological diagnosis of GC confirmed through surgical examination; (2) patients who had contrast-enhanced stomach CT images obtained within 7 days prior to surgery; (3) receiving postoperative adjuvant chemotherapy. Exclusion criteria included: (1) cT1–2 stage,invasion of adjacent organs(cT4b) or distant metastasis (M1 stage) occurred before surgery; (2) poor quality of CT images and little visceral fat, which affected the observation and outlining of peritumour area of ROI; (3) combined with primary malignant tumours in other sites within 2 years; (4) received neoadjuvant chemoradiotherapy or targeted drug therapy before surgery; (5) recurrence of gastric cancer within 1 month after surgery; and (6) death due to other diseases. Finally, a total of 184 cT3–4 GC patients treated between January 2018 and December 2022 were enrolled.

Clinical data, including age and sex, were obtained from the electronic medical records. The cT stage and cN stage were determined by preoperative CT images based on the criteria outlined in the American Joint Committee on Cancer (8th Edition) guidelines for gastric cancer diagnosis (5). The longest diameter and maximum thickness of the gastric cancer were measured on enhanced venous image CT images.The analysis of CT images was performed by two radiologists with 5 and 10 years of experience in GC imaging. In instances where disagreements arose between the two readers, a third radiologist with 30 years of expertise in the field was consulted to review the images and provide a final decision.

All participants were monitored postoperatively, with assessments conducted every three months during the initial year and every six months thereafter. Tumor recurrence was identified through confirmed cases of local or peritoneal recurrence, distant metastasis, or death linked to GC. The presence of recurrence or metastasis was verified using CT, MRI, PET, endoscopic examinations, and laboratory analyses. Based on the follow-up results, all enrolled patients were categorized into two groups: the ER group and the no recurrence group.

### CT examination

All participants maintained a minimum 4-hour fast and received 20 mg of anisodamine via intramuscular administration 10 minutes prior to contrast-enhanced CT imaging to suppress gastrointestinal motility.Patients fasted and consumed 800–1000 ml of water before CT scanning to dilate the stomach. Scans were performed using a 64-slice CT scanner (SOMATON sensation 64, SIEMENS Healthcare, Germany) or 256-slice CT scanner (Brilliance iCT, ROYAL PHILIPS, Netherlands). Parameters included: tube voltage, 120 kVp; tube current, 220–250 mA; detector collimation, 128×0.625 mm or 32×0.6 mm; reconstruction thickness, 5 mm. Scopolamine hydrochloride was administered to reduce gastrointestinal motility artifacts. Contrast agent (ioversol, 320 mg/ml) was injected at 3.0 ml/s, with arterial and venous phase images acquired at 30 s and 70 s delays, respectively.

### Image preprocessing and ROI segmentation

**Figure 1:**
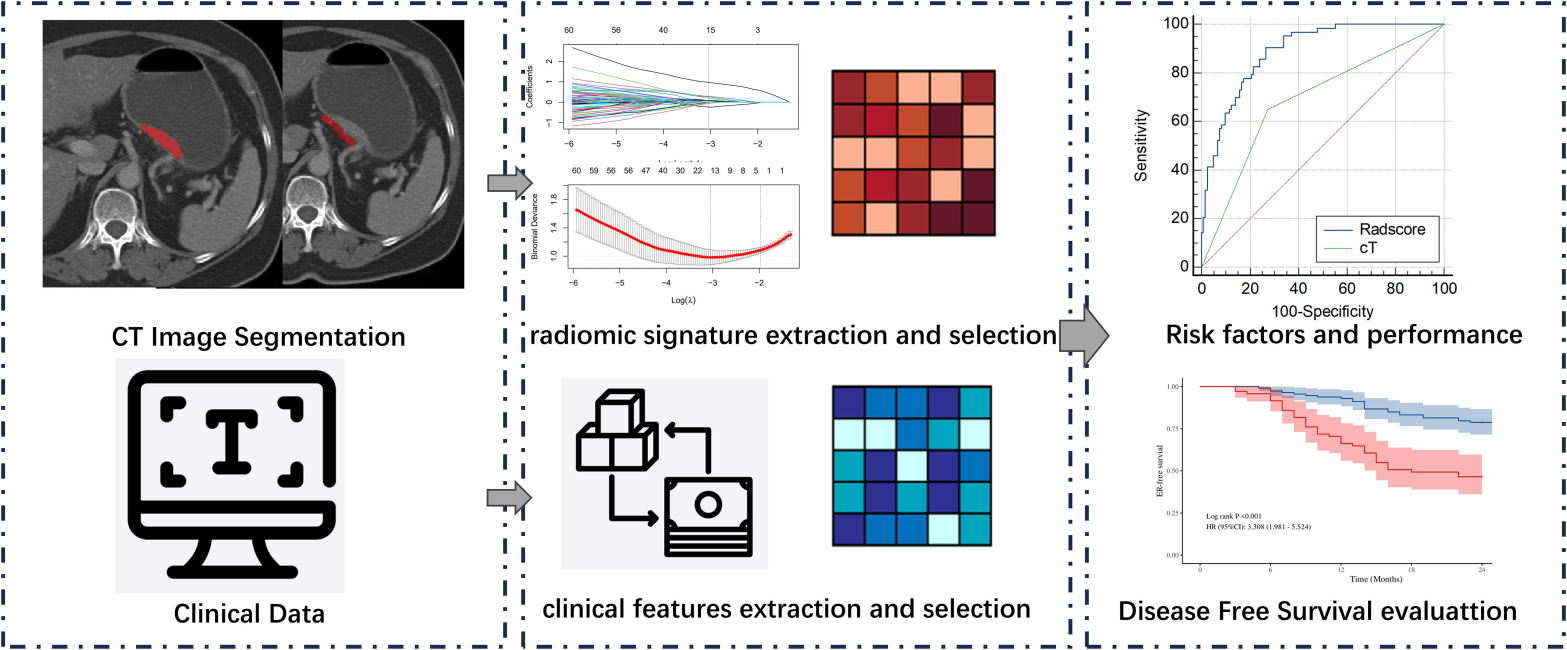
The technical process.

To standardize CT image data from various sources, original DICOM files are converted to NifTI (.nii.gz) format. Image resampling is then applied to ensure consistent voxel size, spacing, and orientation across images from both CT scanners. The CT images were subjected to a downsampling process, resulting in a pixel spacing of 1.0 mm × 1.0 mm × 1.0 mm. This was achieved through the utilisation of a B-spline interpolation algorithm, a technique designed to ensure an isotropic voxel spacing and thereby enhance the reliability of the feature extraction process. Manual segmentation of tumors was performed on venous phase CT images using ITK-SNAP software (version 3.6). The tumor regions of interest (ROIs) should cover the whole volume of the tumor. For the PAT ROIs adjacent to the tumor, radiologists analysed all CT images of the patient and segmented PAT with the largest tumor slice. A strip ROI (width of 5 mm) of PAT was selected along the gastric wall,and should not overlap with regions containing tumor. The technical process of this study is depicted in **Figure 1**. Radiologist A (3 years of experience) delineated the ROIs for the 184 patients in the study and repeated the segmentation procedure 1 month later on 30 patients selected at random. Radiologist B (3 years of experience) subsequently performed the segmentation on the same cases. Segmentations for all patients and the 30 cases were conducted following this methodology, subsequently approved by Radiologist C, with 20 years of experience.

### Radiomics features extraction and selection

The subsequent extraction of radiomic features was then conducted using the PyRadiomics software (version 3.7.16). For each patient, 1130 radiomic features were extracted from CT imaging of tumor and PAT separately. The features thus extracted included: 14 shape features, 18 first-order features, 75 textural features and 1023 high-dimensional features. The high-dimensional features included: 18 first-order features transformed by log-sigmoid, 262 textural features transformed by log-sigmoid, 18 first-order features transformed by wavelet, and 726 textural features transformed by wavelet.

The CT images were subjected to a downsampling process, resulting in a pixel spacing of 1.0 mm × 1.0 mm × 1.0 mm. This was achieved through the utilisation of a B-spline interpolation algorithm, a technique designed to ensure an isotropic voxel spacing and thereby enhance the reliability of the feature extraction process. The subsequent extraction of radiomic features was then conducted using the PyRadiomics software. For each patient, 1130 radiomic features were extracted from CT imaging of tumor and PAT separately. The features thus extracted included: 14 shape features, 18 first-order features, 75 textural features and 1023 high-dimensional features. The high-dimensional features included: 18 first-order features transformed by log-sigmoid, 262 textural features transformed by log-sigmoid, 18 first-order features transformed by wavelet, and 726 textural features transformed by wavelet.

To assess the stability and reproducibility of radiomics features, we evaluated inter-observer consistency using the intraclass correlation coefficient (ICC). A subset of 30 randomly selected samples underwent re-segmentation and feature re-extraction. Features demonstrating good consistency (ICC > 0.75) were retained for subsequent analysis.

All intratumoral and PAT radiomic features underwent Z-score normalisation to reduce dimensional heterogeneity between radiomic indices. In addition, features were compared between ER and ER-free groups using the Mann-Whitney U test, with insignificant (p ≥ 0.05) features removed. Feature selection was performed utilizing LASSO regression, which applies L1 regularization to shrink the coefficients of uncorrelated features to zero, thereby automatically selecting features. The optimal regularization parameter (λ) was selected through 10-fold cross-validation to enhance model performance. As λ increases, coefficients of uncorrelated features gradually diminish, and the optimal λ value (λ_min) that maximizes model fit was identified based on the log-likelihood ratio. Features associated with non-zero coefficients were preserved. The radscore for each patient was calculated from the selected radiomics features and their coefficients, with an intercept (α) of -.54975188, as previously described (10).

### Tatistical analysis

Clinico-radiology features and radscores were compared between patients with and without ER. Chi-squared tests were employed to compare categorical data between groups. Continuous data were analyzed using either the t-test or the Mann–Whitney U test, contingent on normality and variance homogeneity.To identify independent prognostic factors, we first used univariable Cox proportional hazards regression analyses to evaluate the association between variables and ER-free survival. Subsequently, due to the constrained sample size, bivariate Cox regression analyses were conducted by sequentially pairing each univariately significant variable (p<0.05).Independent risk factors for ER were analyzed using receiver operating characteristic (ROC) curves, with the area under the curve (AUC) calculated. The DeLong test was used to evaluate differences between these independent risk factors with MedCalc software (version 23.0; MedCalc Software Ltd, Ostend, Belgium).Based on the median, continuous variables were categorized into high-risk and low-risk groups.Finally, ER-free survival curves were constructed using the Kaplan-Meier method and the log-rank test was used for comparisons between groups.Analyses were performed using R (version 3.6.0) or SPSS (version 26.0). A p-value < 0.05 was considered significant.

## Results

### Clinico-radiologic characteristics

Of the 184 LAGC patients who were enrolled in the study, 34.24% (63/184) experienced postoperative recurrence within two years. Of the patients who experienced recurrence, 25 had local recurrence, 21 had distant recurrence, and 6 had a combination of local and distant recurrence. The median interval to recurrence was 12.30 ± 5.36 months. Tumor location, thickness,cT stage, cN stage and radscore exhibited significant differences between the ER-free patient group and the ER patient group.A detailed description of the GC patients clinical and CT imaging features is provided in **Table 1**.

**Table 1:** Comparison of clinical and CT imaging features between the early recurrence and no recurrence groups.

Variables	Total (n = 184)	No recurrence (n = 121)	Early recurrence (n = 63)	*P*
Age, M (Q_1_, Q_3_)	66.00 (59.00, 71.00)	66.00 (59.00, 71.00)	65.00 (58.50, 70.00)	0.618
Sex (male: female)	139:45	91:30	48:15	0.883
Location (Esophagogastric junction: Gastric antrum and body)	89:95	52:69	37:26	**0.042**
Longest diameter, M (Q_1_, Q_3_)	5.70 (4.50, 7.12)	5.50 (4.50, 7.00)	6.00 (4.85, 7.35)	0.177
Maximum thickness, M (Q_1_, Q_3_)	1.39 (1.00, 1.80)	1.22 (1.00, 1.60)	1.50 (1.15, 1.95)	**0.012**
cT stage(T3:T4)	110:74	88:33	22:41	**<.001**
cN stage (N0:N1-3)	44:140	37:84	7:56	**0.003**
Radscore, M (Q_1_, Q_3_)	-0.60 (-1.24, -0.01)	-1.08 (-1.36, -0.48)	0.06 (-0.24, 0.30)	**<.001**

The bold values indicate statistical significance.

### Radiomics feature selection and radscore calculation

**Figure 2:**
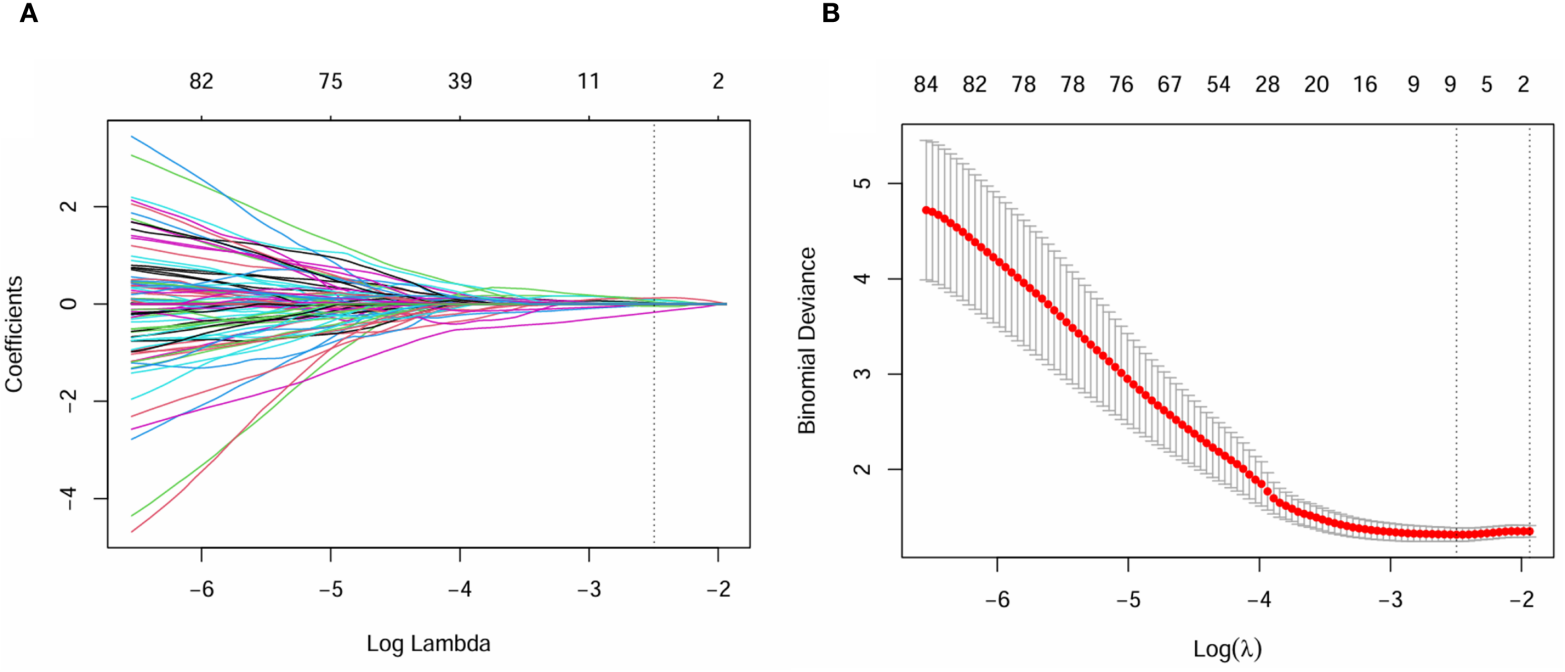
Least absolute shrinkage and selection operator (LASSO) coefficient profiles of the radiomics features, Panel **(A)** displays the selection of the tuning parameter (λ) via cross-validation, while Panel **(B)** shows the coefficient profile plot, demonstrating variations in feature coefficients with changes in the regularization parameter λ. Features that are retained exhibit non-zero coefficients.

Inter-observer consistency analysis revealed a median ICC value of 0.75 across all radiomic features, indicating good agreement between the two operators in feature delineation. Out of the 2260 extracted radiomics features, 1513 features exhibited an ICC greater than 0.75.Of these, a subset of 762 were deemed different in ER and ER-free groups using the Mann-Whitney U test.Following the exclusion of non-reproducible and redundant features, 2 tumour-related radiomic features and 3 PAT-related radiomic features with non-zero coefficients were identified by LASSO regression algorithm (**Figure 2**). These features included 1 Shape features and 2 wavelet transformed texture features from PAT, 2 Filter-Based Texture Features from intratumor (**Table 2**). The radscore for each patient was determined using the formula described above.

**Table 2:** Selected radiomics features.

Radiomics features	Origin	Coefficient
original_shape_SurfaceVolumeRatio	peritumoral adipose tissue	0.54650564
wavelet.LHL_glrlm_RunEntropy	peritumoral adipose tissue	-0.08031735
wavelet.HHL_glrlm_RunVariance	peritumoral adipose tissue	-0.16353140
exponential_glrlm_RunLengthNonUniformity	intratumor	0.02197938
square_glszm_SizeZoneNonUniformity	intratumor	0.13748354

### Risk predictors of ER and stratified survival analysis

**Figure 3:**
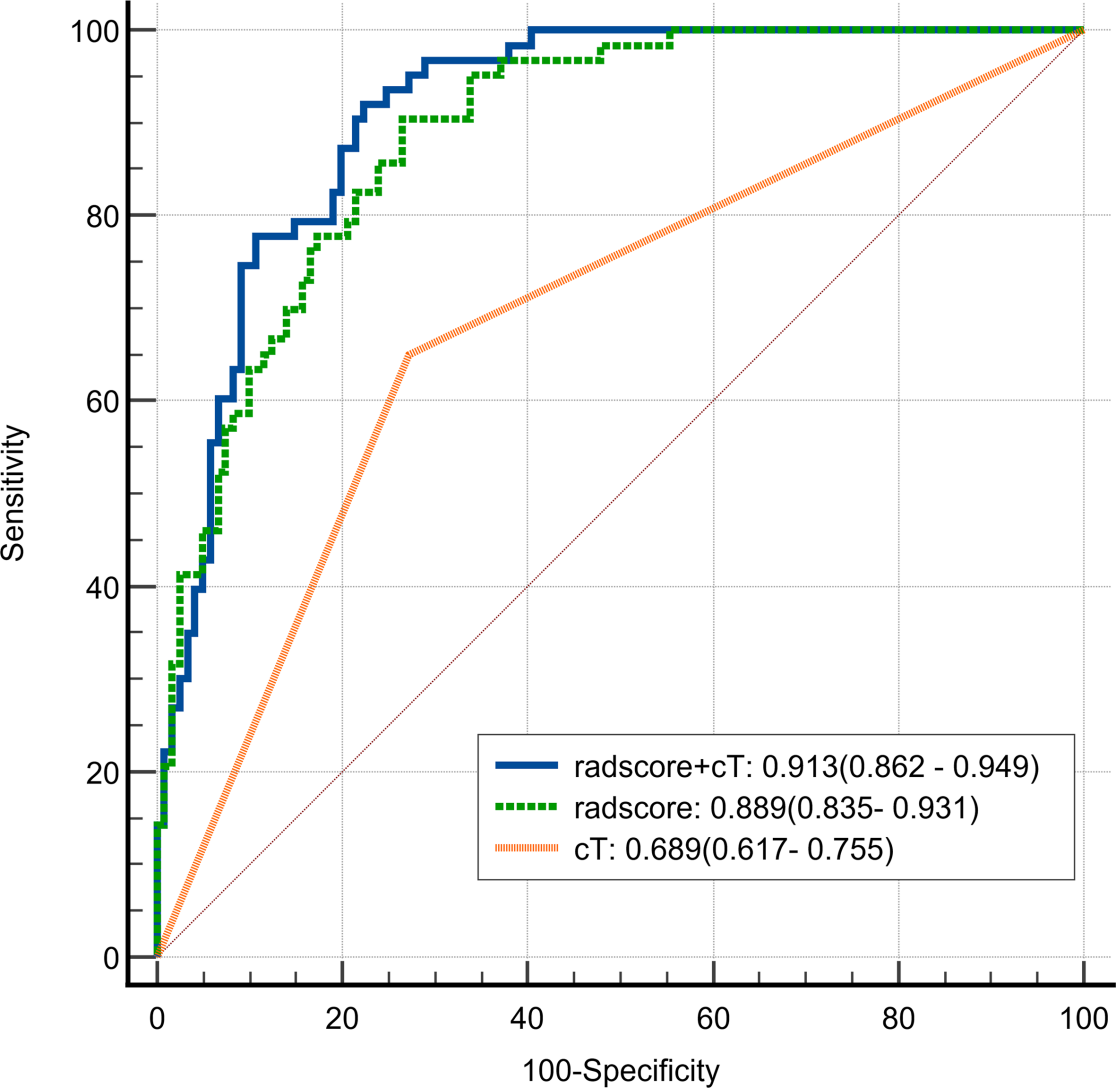
Receiver Operating Characteristic (ROC) curve of radscore plus cT, radscore alone, and cT alone in differentiating early recurrence in patients with cT3–4 gastric cancer.

Univariate Cox regression analysis revealed that larger tumor longest diameter (HR, 1.04; 95% CI, 1.01 ~ 1.09; p =0.035), larger tumor maximum thickness (HR, 1.10; 95% CI, 1.01 ~ 1.20; p =0.030),cT 4 stage(HR, 3.98; 95% CI, 2.35 ~ 6.75; p <0.001), cN1–3 stage (HR, 3.28; 95% CI, 1.41 ~ 7.62; p =0.006) and higher radscore (HR, 5.71; 95% CI, 3.71 ~ 8.79; p <0.001) were identified as significant predictors for ER (**Table 3**). Bivariate Cox regression analyses demonstrated that cT stage and radscore emerged as independent predictors of ER in all adjusted model configurations(**Table 4**). To evaluate the predictive performance of the two independent predictors—cT stage and radscore—along with their combination, ROC curve analysis was performed (**Figure 3**), using the area under the curve (AUC) as the key metric of discriminative ability. According to the DeLong test, the combined model (cT stage + radscore) significantly outperformed both the cT stage alone and the radscore alone. Furthermore, the radscore alone showed a statistically higher AUC than the cT stage.

**Table 3:** Univariate Cox regression analysis for early recurrence free survival.

Variables	*P*	Hazards ratio	95% confidence interval
Age	0.853	1.00	0.97 ~ 1.03
Sex	0.662	0.88	0.48 ~ 1.59
Location	0.055	0.61	0.37 ~ 1.01
Longest diameter	**0.035**	1.04	1.01 ~ 1.09
Maximum thickness	**0.030**	1.10	1.01 ~ 1.20
cT stage	**<.001**	3.98	2.35 ~ 6.75
cN stage	**0.006**	3.28	1.41 ~ 7.62
Radscore	**<.001**	5.71	3.71 ~ 8.79

The bold values indicate statistical significance.

**Table 4:** Bivariate Cox regression analysis for early recurrence free survival.

No.	Variables	*P*	Hazards ratio (95% confidence interval)		Variables	*P*	Hazards ratio (95% confidence interval)
1	cT stage	**<.001**	3.39 (1.96 ~ 5.88)	6	cN stage	**0.008**	3.15 (1.35 ~ 7.33)
	cN stage	0.117	2.02 (0.84 ~ 4.85)		Maximum thickness	0.083	1.09 (0.99 ~ 1.19)
2	cT stage	**<.001**	3.86 (2.26 ~ 6.57)	7	cN stage	0.096	2.06 (0.88 ~ 4.81)
	Longest diameter	0.268	1.02(0.98 ~ 1.07)		Radscore	**<.001**	5.45 (3.51 ~ 8.45)
3	cT stage	**<.001**	3.88 (2.28 ~ 6.59)	8	Longest diameter	0.661	1.02 (0.93 ~ 1.12)
	Maximum thickness	0.168	1.07 (0.97 ~ 1.17)		Maximum thickness	0.607	1.06 (0.86 ~ 1.30)
4	cT stage	**0.002**	2.43 (1.40 ~ 4.22)	9	Longest diameter	**0.035**	1.04 (1.01 ~ 1.09)
	Radscore	**<.001**	4.61 (3.00 ~ 7.08)		Radscore	**<.001**	5.78 (3.74 ~ 8.92)
5	cN stage	**0.007**	3.17 (1.36 ~ 7.38)	10	Maximum thickness	**0.030**	1.10 (1.01 ~ 1.21)
	Longest diameter	0.078	1.04		Radscore	**<.001**	5.79 (3.75 ~ 8.94)

The bold values indicate statistical significance.

**Figure 4:**
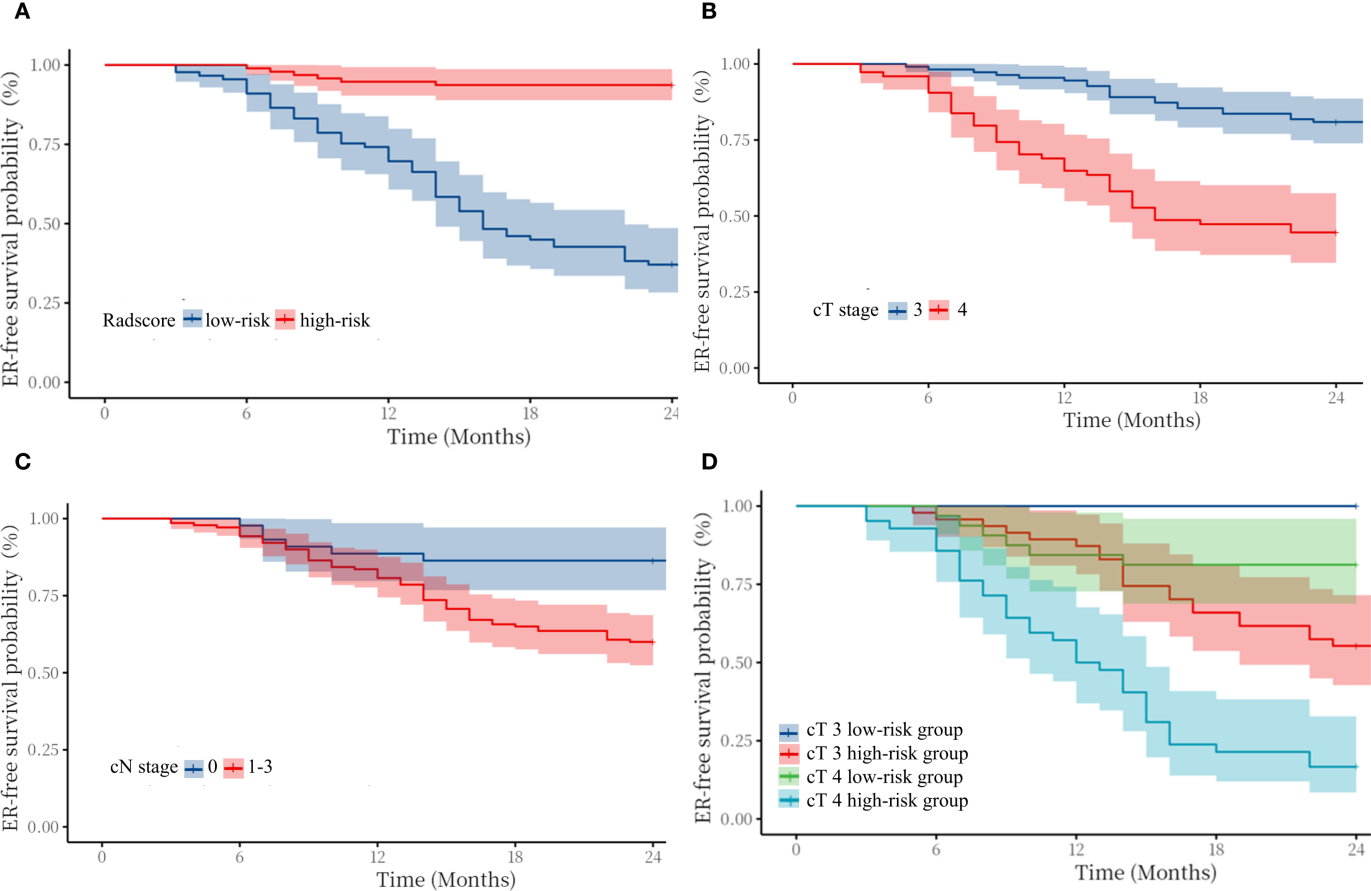
Kaplan–Meier survival curves for early recurrence in patients with cT3–4 gastric cancer stratified by radscore **(A)**, cT stage **(B)**, cN stage **(C)**, and cT3–4 stage in the high/low-risk category **(D)**.

The median value of radscore was -0.60. The Kaplan–Meier (**Figure 4**) curve showed that patients in the high -risk category (radscore ≥-0.60) exhibited a higher ER rate compared to those in the low -risk category (64.0% vs 6.3%, log-rank test: p < 0.001). Patients with cT4 stage exhibited a higher rate of ER compared to those with cT3 stage (53.5% vs 22.1%, log-rank test: p < 0.001). Patients with cN1–3 stage exhibited a higher rate of ER compared to those with cN0 stage (40.0% vs 15.9%, log-rank test: p = 0.003).

In addition, we compared the ER-free survival of cT3 patients with cT4 patients in different risk groups, stratified according to radscore. The survival curves (**Figure 3**) showed that cT3–4 patients in low-risk group exhibited better prognosis than the same cT stage patients in high-risk group. Notably, the cT3 patients in high-risk group exhibited worse prognosis than the cT4 stage patients in low-risk group(log-rank test: p < 0.01). **Figure 4** shows typical cases images.

## Discussion

The present study investigated preoperative prognostic factors derived from CECT for ER in patients with cT3–4 GC. The findings of the study revealed that both the radscore and cT stage are non-invasive imaging markers for preoperatively predicting ER in GC patients; Particularly, the tumor and PAT derived radscore might improve the accuracy of risk stratification, which coulld be applied to guide gastric cancer patients to receive precise and personalised treatment.

The cT staging are important prognostic factors in patients with GC (11, 12).The present findings also suggest that GC with serosal penetration (cT4) have a higher recurrence risk than those with subserosal infiltration (cT3).When gastric cancer invades the serosa, PAT is the most likely site for metastatic spread.The ‘seed and soil’ theory proposes that peritoneal metastasis initiation depends on the synergies of the tumor cells (seeds) and the peritoneal microenvironment (soil) (13). Conventional CT-based T-staging relies on subjective morphological interpretation of tumour infiltration depth. A review (14) reported the diagnostic accuracy of CT for distinguishing stage T4 from non-T4 stages varies from 68% to 80%.Conventional CT has limited discriminative capacity in differentiating neoplastic infiltration from paraneoplastic inflammatory responses. This is due to overlapping radiographic manifestations, characterised by increased attenuation within PAT (15). Previous studies have shown that dual-energy CT-derived iodine quantification within PAT has been validated as a reliable indicator for detecting serosal invasion in gastric cancer (16, 17). Elevated iodine concentrations in PAT reflect enhanced perfusion due to tumor-induced serosal disruption, potentially mediated by neovascularization or malignant cellular membrane leakage. However, even though CT-defined depth of tumour invasion is not completely consistent with pathological findings, the cT staging is a critical prognostic determinant in the management of gastric cancer, which is in agreement with the results of the previous study (18).The cN staging of GC is of significant prognostic importance for overall survival (19, 20), The present univariate analyses revealed a significant association of cN staging with ER. However, the cN staging lost independent prognostic significance following bivariate adjustment for cT staging and radscore. This lack of significance likely stems from CT’s limited diagnostic accuracy in detecting metastatic lymph nodes, which rangers from 51%–58% by CT (21). Consequently, the suboptimal results observed in the radiological assessment of lymph nodes undermined their prognostic value, which is similar to the results of previous studie (22).

The metastatic outgrowth of tumors arises from dynamic interactions and mutual adaptation between tumor cells and the surrounding microenvironment (23). In the present study, the strong performance of the radscore may be attributed to its integration of CT-based tumor and PAT radiomic features. By leveraging automated high-throughput feature extraction, radiomics deciphers tumor heterogeneity patterns, enabling robust predictions of recurrence-free survival outcomes (7). An increased original_shape_SurfaceVolumeRatio from PAT signifies irregular and complex morphology, often associated with invasive growth and poor differentiation, indicative of aggressive behavior. Multiple textural characteristics collectively indicate significant intratumoral and peritumoral heterogeneity, including wavelet-based RunEntropy and RunVariance from tumor, exponential RunLengthNonUniformity, and square SizeZoneNonUniformity from PAT. These features collectively suggest heightened randomness, diverse spatial scales, and uneven distribution of texture patterns. This heterogeneity likely arises from underlying biological processes such as necrosis, hemorrhage, vascular proliferation, stromal infiltration, fibrosis, and unevenly dispersed tumor subclones. Overall, these characteristics quantitatively capture the morphological and textural intricacy linked to aggressive tumor behavior, offering potential non-invasive biomarkers for assessing tumor progression and proliferative activity. Notably, the increased quantity and biological relevance of peritumoral features underscore the pivotal role of the tumor microenvironment in disease advancement. In hepatocellular carcinoma, it was reported that a peritumoural radiomics model that included 2 cm of peritumour on CT was more accurate in predicting early recurrence than tumour models (AUC 0.79 vs. 0.62) (24). In non-small cell lung cancer, it was reported that a peritumoural radiomics model that included 10 mm of peritumour on CT exhibited best predictive efficiency for predicting spread through air spaces (25). As a quantitative method, radscore allows the quantification of intratumoral heterogeneity and variations in the peritumoral microenvironment.Furthermore, CT-based radscore enabled risk stratification of cT3 and cT4 GC subgroups, significantly enhancing prognostic discrimination. The cT3 staging tumors with high-risk radscores exhibited inferior survival outcomes relative to cT4 staging lesions harboring low-risk signatures.Our findings suggest that radscore could assist in stratifying patients for neoadjuvant therapy. In addition, GC cases with high-risk radscore may require more intensive postoperative surveillance.

This study has some limitations. Firstly, the study was conducted retrospectively at a single centre, and the sample size was relatively small. Therefore, it should be considered as a pilot study, which deserves to be confirmed by larger scale studies to confirm the results. Secondly, the study population consisted of cT3–4 staging GC patients receiving postoperative adjuvant chemotherapy, which may have introduced a degree of bias. The ability of radscore needs to be evaluated in further studies involving all GC patients.Thirdly, the relatively short follow-up period may have resulted in incomplete survival information for some patients.

## Conclusions

This retrospective study demonstrates that contrast-enhanced CT-derived radscore incorporating tumor and PAT features serve as effective preoperative predictors of ER in cT3–4 GC patients, highlighting their potential to refine prognostic stratification and optimise clinical decision-making through personalised therapeutic approaches and surveillance protocols.

## Data Availability

The original contributions presented in the study are included in the article/Supplementary Material. Further inquiries can be directed to the corresponding author.
